# Polarized Light Sensitivity and Orientation in Coral Reef Fish Post-Larvae

**DOI:** 10.1371/journal.pone.0088468

**Published:** 2014-02-07

**Authors:** Igal Berenshtein, Moshe Kiflawi, Nadav Shashar, Uri Wieler, Haim Agiv, Claire B. Paris

**Affiliations:** 1 Department of Life Sciences, Ben-Gurion University of the Negev, Eilat Campus, Beer-Sheva, Israel; 2 The Interuniversity Institute for Marine Sciences of Eilat, Eilat, Israel; 3 Department of Animal Sciences, The Hebrew University of Jerusalem, Rehovot, Israel; 4 Applied Marine Physics, Rosenstiel School of Marine and Atmospheric Science, University of Miami, Miami, Florida, United States of America; Lund University, Sweden

## Abstract

Recent studies of the larvae of coral-reef fishes reveal that these tiny vertebrates possess remarkable swimming capabilities, as well as the ability to orient to olfactory, auditory, and visual cues. While navigation according to reef-generated chemicals and sounds can significantly affect dispersal, the effect is limited to the vicinity of the reef. Effective long-distance navigation requires at least one other capacity–the ability to maintain a bearing using, for example, a sun compass. Directional information in the sun’s position can take the form of polarized-light related cues (i.e., e-vector orientation and percent polarization) and/or non-polarized-light related cues (i.e., the direct image of the sun, and the brightness and spectral gradients). We examined the response to both types of cues using commercially-reared post-larvae of the spine-cheeked anemonefish *Premnas biaculeatus*. Initial optomotor trials indicated that the post-larval stages are sensitive to linearly polarized light. Swimming directionality was then tested using a Drifting In-Situ Chamber (DISC), which allowed us to examine the response of the post-larvae to natural variation in light conditions and to manipulated levels of light polarization. Under natural light conditions, 28 of 29 post-larvae showed significant directional swimming (Rayleigh’s test p<0.05, R = 0.74±0.23), but to no particular direction. Swimming directionality was positively affected by sky clarity (absence of clouds and haze), which explained 38% of the observed variation. Moreover, post-larvae swimming under fully polarized light exhibited a distinct behavior of tracking the polarization axis, as it rotated along with the DISC. This behavior was not observed under partially-polarized illumination. We view these findings as an indication for the use of sun-related cues, and polarized light signal in specific, by orienting coral-reef fish larvae.

## Introduction

Animals are often required to navigate a straight course, either towards or away from a particular location [1], [2]. To achieve this feat they usually rely on an external reference; or compass [3]. A compass can also facilitate reorientation following unintended displacement, or bridge spatial and/or temporal discontinuities in a stimulus (e.g. chemical or acoustic) they wish to follow. Clearly, a guiding compass would benefit most actively mobile organisms; including the pelagic larvae of coral reef fishes. For these dispersive elements, reaching an unobservable reef on which to settle is a matter of life or death; with far-reaching demographic and ecological consequences [4].

Despite their minute size, coral-reef fish larvae are known to be effective swimmers [5]. Moreover, they have been shown to possess sensory capabilities that could locate distant targets for settlement (olfactory: e.g. [6], [7], [8], [9]; auditory: e.g. [10], [11]). Together, these capacities afford larvae with the potential to actively affect their dispersal [12], [13]. However, the effective range of the sensory capabilities is largely unknown; as is the capacity for compass-orientation, which would render larvae effective navigators.

Compass-orientated swimming by coral-reef fish larvae has been suggested in several studies [14], [15], [16], [17]. It has been tested for settlement stages in the laboratory using a clock-shift experiment [18] and in the field using in-situ manipulation (Paris CB, Irisson JO, Leis JM, Boguki D, Piskozub D, et al., unpublished data). The present study is an extension of the latter, with the ultimate goal of testing the effect of polarized-light on larval swimming directionality. The test-subject of the study is of post-larval *Premnas biaculeatus* (Pomacentridae).

The use of the sun as a frame of reference (sun compass) is well documented (e.g. [19], [20], [21]). Directional information is afforded directly by the sun’s position, but also indirectly by patterns of light polarization [22], [23]. Several researchers suggested three modes by which directional information can be conveyed by linearly polarized light, underwater [24], [25], [26], [27]. 1) Polarization patterns in the sky, as refracted through Snell’s window [27]. 2) The percent polarization and e-vector orientation, resulting from scattering of refracted light in the water [24], [25]. 3) Differences in polarization patterns between deep and shallow waters [25], [28]. The depth to which the polarized-light signal penetrates depends on many factors; such as: water clarity, wave action, bottom depth, and the sun’s elevation over the horizon. None the less, *in-situ* measurements have shown sun-related partial polarization reaching down to 200 m [29]; well below the depths occupied by most coral-reef fish larvae (e.g. [30], [31]). However, the percent of linear polarization in the ocean rarely exceeds 60% [26], [32].

Sensitivity to polarized light need not be limited to orientation purposes, and can play an important role in prey and predator detection [33]. In coral-reef damselfish, sensitivity to polarized light has been demonstrated both physiologically, using electroretinogram [34], and behaviorally, using fish trained to swim relative to e-vector orientation [35]. Below we present evidence for polarized-light sensitivity in *P. biaculeatus*, based on a modification of the classic optomotor-response experiments [36], [37]. In addition, we present results from field experimentation involving a “Drifting-In-Situ-Chamber” (DISC) [38], which shows that post-larval fish can enhance their swimming directionality using both polarized and non-polarized-light cues related to the sun’s position.

## Materials and Methods

### Ethics Statement

This study was approved by the committee for the Ethical Care and Use of Animals in Research of the Ben-Gurion University (Permit Number: IL-77-12-2011), and by the Israel Nature and National Parks Protection Authority (Permit Number: 2012/38383). All efforts were made to minimize animal suffering.

### Study Organism

We used commercially reared larvae of the *Premnas biaculeatus* (Pomacentridae), which geographic range covers the Indo-Malayan Archipelago and northern Great Barrier Reef [39]. Larvae were obtained from Ardag inc. (Eilat, Israel), where they were reared in a recirculating sea-water system at a constant temperature (28.0±0.2°C) and salinity (30‰). At the age of 4 DPH (days post hatching), larvae were transferred to a new tank in which the original rearing water was gradually replaced by fresh ambient sea water (salinity of 40‰ and sea temperature of 22±0.2°C). To prevent mechanical damage during transportation to the test site, larvae were shipped in a 1L transparent plastic container placed inside a second 10 L water-filled container. At the site, the larvae were kept inside an aerated 10 L tank, and fed daily with brine-shrimp *(Artemia salina)* nauplii. The age of specimen used in the experiments was 16–23 DPH. For these, pigmentation (metamorphosis) occurred at the age of ∼16 DPH, thus they are considered post-metamorphosis (post-settlement stage) larvae. Total length of the post-larvae was 6–9 mm.

### Laboratory Experiment: Optomotor

**Figure 1:**
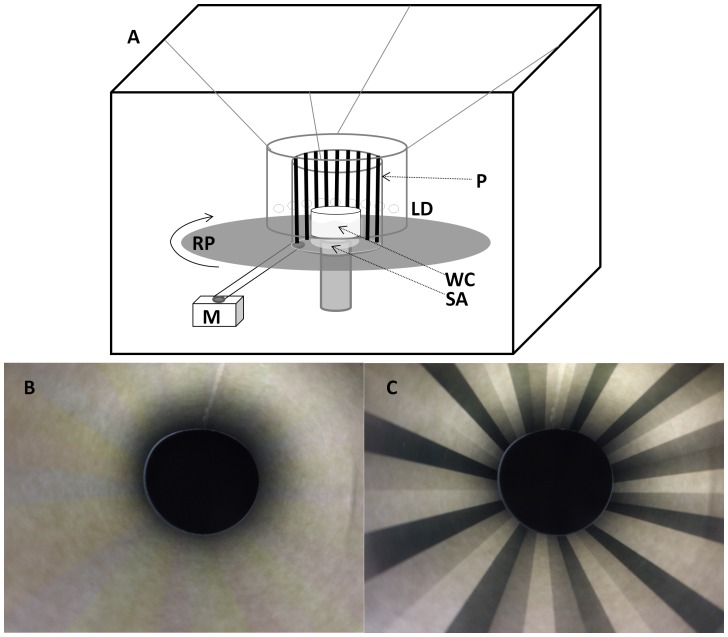
The optomotor apparatus. (A) The optomotor consisting of: a water container (WC), a stationary arena (SA), a rotating platform (RP), an electric motor (M), an experimental pattern (P), light emitting diodes- LEDs (LD). (B) The polarized experimental pattern photographed from the stationary arena looking upwards, (C) same image as B, but viewed through a polarizing filter.

Classic optomotor response experiments rotate a vertically-striped black and white pattern around a test subject, which is placed inside a stationary arena. To the extent that the subject discerns the rotating pattern it will attempt to follow it in order to stabilize movement in its visual field, i.e. to follow large scale movements of its visual scene (optometric response; [36]). The optomotor apparatus used for this experiment (Fig. 1A) was used previously in [37], [40], and is a slightly modified version of the one used in [41]. Briefly, the apparatus consists of a cylinder with controllable rotation speed and direction (clockwise or counterclockwise). To test for polarization sensitivity, the black and white stripes are replaced with stripes of different e-vector orientations. Since the rotating pattern is discernible only by subjects with polarized-light sensitivity, a positive swimming response would validate the putative sensitivity.

We used three different rotating patterns (treatments): 1) BW- 9 mm black and white vertical stripes, as a positive control; 2) POL- polarized 9 mm vertical stripes with e-vector orientations of 0 (horizontal), 45, 90 and 135 degrees; 3) W- a white sheet with no pattern on it, as a negative control. Each of the patterns was placed inside a thin plastic diffusing-white cylinder, acting as a diffuser as well as for keeping pattern round. A circular glass dish (diameter = 12 cm) filled with sea water was placed on the central stationary platform. Illumination was provided by three strips of 48 white LEDs (light emitting diodes), glued on an opaque cylinder (diameter = 26.5 cm) that encompassed the rotating pattern. A diffuser sheet was attached in front of LEDs to insure the depolarization of the illumination source. The LEDs were set at the same height as the aquaria to prevent reflections on the experimental pattern. Such reflections could reveal the polarized striped pattern to polarization insensitive viewer [42]. As seen in Fig. 1B, the light intensity in the POL experimental pattern was homogenous and did not reveal the striped pattern due to reflection or differential light intensity. A video camera (Sony, DCR-PC110E) mounted above the dish enabled real-time monitoring and recording of the larva’s behavior.

We examined 59 post-larvae, which ranged in age from 16 to 23 days. Test subjects were allowed 5 minutes of acclimation, in the central arena, prior to experimentation. Each individual was then observed under all three treatments; offered at random order. Each treatment was offered for 1 minute per direction of rotation (clockwise (CW) and anti-clockwise (CCW)). Initial rotation direction was chosen at random. The speed of rotation was increased gradually (during approx. 2 seconds) up to the experimental speed of 12.5 RPM. Preliminary trials with the BW pattern showed that the optomotor response of *P. biaculeatus* post-larvae peaked at this speed (range of speeds examined 0–20 RPM: n = 20).

Larval response to each treatment was scored positive if it followed the direction of rotation for at least one complete circle (360°) on both directions (CW and CCW). If the post-larva failed to do so, it was scored negative response. An individual was scored “polarization sensitive” only if it scored positively for both BW and POL, and negatively for the W control. Cases, in which the response of the larva was hard to be determined (positive or negative), were listed as “borderline” and were treated separately (see results section).

### Field Experiment: Drifting In-Situ Chamber (DISC)

Experimentation using the DISC [38] (https://www.rsmas.miami.edu/users/cparis/instruments.html) followed one of two approaches. First- the use of natural variation in environmental variables to explain the observed variation in their swimming directionality (R; the degree of oriented swimming calculated as the mean resultant vector of the sampled angles). Directionality was computed both at the “individual level” (using the positions of each larva in the behavioral chamber) and “among individuals level” (using mean bearings of the larvae). Second - manipulation of percent-polarization and the direction of the polarization axis, to test their influence on swimming directionality. Both are expanded upon below, following a brief description of the DISC and its deployment.

**Figure 2:**
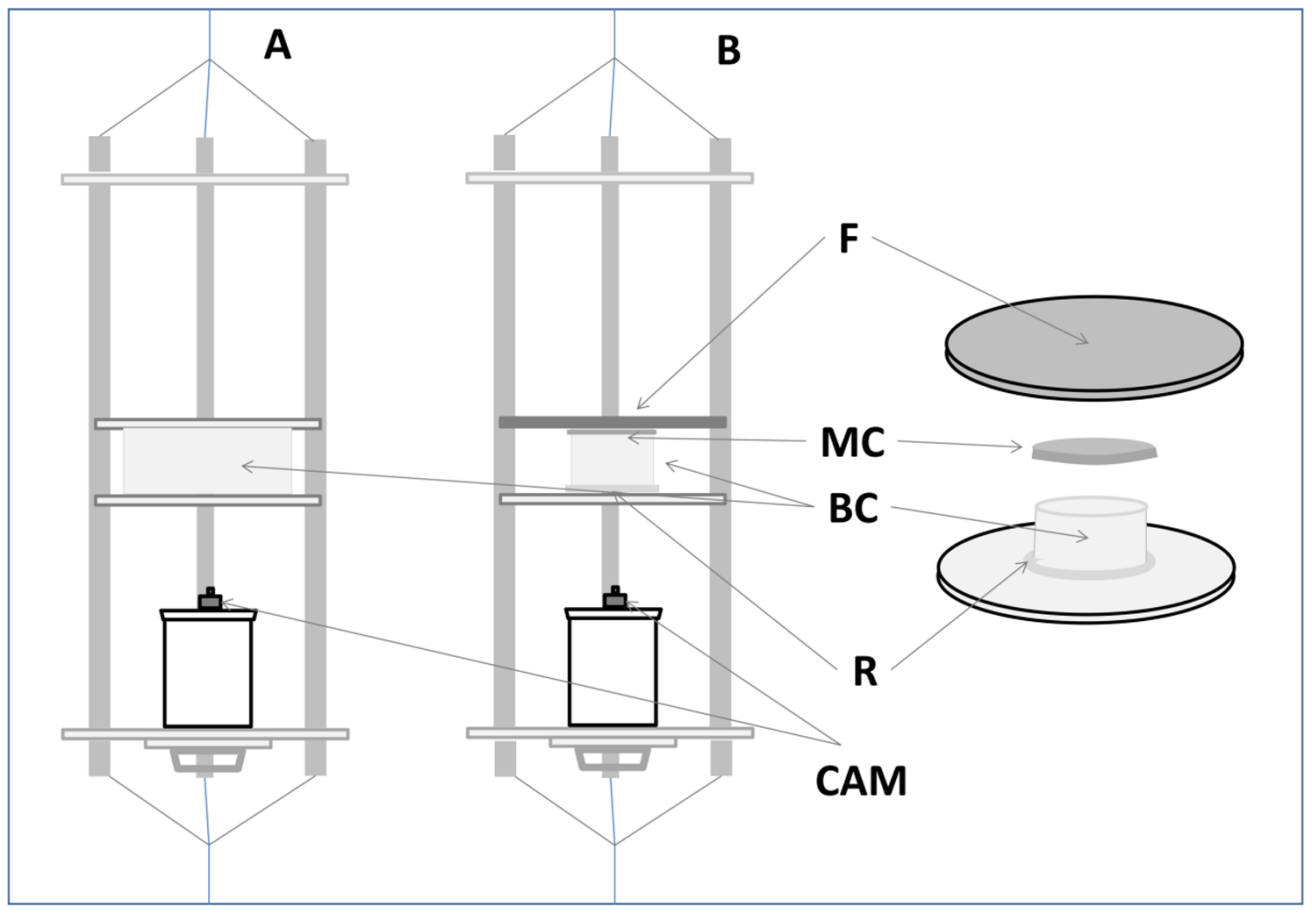
The DISC: Drifting In-Situ Chamber. The DISC is a cylindrical transparent and symmetrical behavioral chamber, which is set adrift and records the behavior of individual larvae (https://www.rsmas.miami.edu/users/cparis/instruments.html). (A) The classic DISC setup used in the natural conditions (NC) experiment consisting of a large behavioral chamber. (B) The setup used for the polarization manipulation experiment consisting of: the three layered filter (F), the mesh cover (MC), the behavioral chamber (BC), the acrylic ring (R), and the camera (CAM).

The DISC [15], the corresponding image analysis and statistical software package [17] used in this study differed slightly from earlier versions and is detailed in [38]. Briefly, it is a cylindrical symmetrical structure equipped with a transparent behavioral chamber and a data recording system (Fig. 2A) connected with a thin line to a surface buoy. A Global Positioning System (GPS) logger (I-gotU GT 120, Mobile Action Technology) connected to the float provides continuous recording of the DISC’s position and drift. A transparent drogue with a 4 pound weight is attached at the bottom of the DISC, to keep the DISC locked with the current at the deployment depth. Since the DISC is embedded in the water mass, the position displacement of the DISC over time represents the speed of the current which the DISC is drifting in [38]. The main difference between the current and latest version include: the addition of a bottom-mounted GOPRO Hero2 (Woodman labs) camera replacing a Nikon SLR D70 camera and a smaller behavioral arena.

We used one of two behavioral chambers. For observations made under natural light conditions (NC) we used the large chamber (Fig. 2A; diameter: 38 cm, hight: 12 cm), made entirely from transparent 1 mm nylon mesh [15]. Here, a small (diameter: 10 cm) transparent Petri dish, fixed to the bottom of the chamber, enabled safe introduction of non-native test subjects into the chamber while it was suspended on a davit above the water surface. A smaller chamber (Fig. 2B; diameter: 16 cm) was used for the polarization manipulation. The bottom of this chamber was constructed by gluing a small acrylic ring (inner diameter: 16 cm, outer diameter: 18 cm, height: 2 cm), to the middle of a circular acrylic bottom plate (Fig. 2B; diameter: 44 cm), which acted as a small seawater reservoir for the larva during DISC deployment (same function as the petri dish in NC trials). A transparent 1 mm nylon mesh was attached at the inner wall of the acrylic ring, acting as the chamber’s side wall (height: 10 cm). The chamber was ‘capped’ by a circular 1 mm nylon mesh lid (Fig. 2B; diameter: 16.3 cm, side-wall height: 1.5 cm), which facilitated the introduction and removal of the specimen.

The DISC was deployed from a motor boat to a depth of 9 m, 500 m offshore from the Interuniversity Institute for Marine science in Eilat (IUI. 29°30.07′ N 034°55.02′ E). DISC deployment and retrieval followed a similar procedure to the one described in [38]. Each deployment lasted 15 min, the first 5 of which served for acclimation and were not analyzed. Time-lapse photos of the behavioral arena were taken at 2 s intervals, throughout the deployment. In total we analyzed 89 deployments: 43 under natural conditions, 20 with partial polarization and 26 with full polarization. Field work spanned Feb–March, 2012.

### Natural Variation in Environmental Variables

Several variables were considered as potential predictors of swimming directionality under natural light conditions; including: sun elevation and azimuth, overcast conditions, bottom-depth, wind and current velocity (which were resolved to long- and cross shore components), and larval age.

Wind velocity and solar radiation were obtained from the meteorological station of the Israel National Monitoring Program (http://www.meteo-tech.co.il/eilat-yam/eilat_en.asp); located at the IUI. Overcast condition during deployments (including cloud cover and haze) was quantified by an index of “normalized solar radiation” (NSR): the solar radiation during deployment, expressed as a proportion of the radiation measured at the same time of day of a perfectly clear day. The two readings (experimental and reference) were separated by no more than 10 days. NSR served as a good proxy for the degree of which the sun was visible during the deployments (Spearman Rank correlation, R = 0.783, p<0.01, details in Text S1, Fig. S1).

### Polarized-light Manipulation

For polarization manipulation we used a three-layered filter, placed approximately 2 mm above the mesh lid of the behavioral chamber; immediately following the positioning of the larvae (Fig. 2B). The filter consisted of a polarizer (Polaroid HN38S, diameter: 40 cm) and a diffuser (commercially used white matte plastic sheet, diameter: 40 cm), glued on opposite sides of a round polarization inert acrylic plate (diameter: 44 cm). This set-up covered Snell’s window from all points within the 10 cm height circular chamber (Fig. 2B), and modified the e-vector orientation such that dowelling light was linearly polarized with the same e-vector as the polarization axis of the filter.

Percent polarization of downwelling light transmitted through the filter depended on whether the polarizer or the diffuser were facing down, creating 95% and 48% linear polarization, respectively (details about the calculation of percent polarization in Text S1). Notably, the level of partial polarization is below the threshold level for detection described for other teleosts (65–75% for rainbow trout [43], [44]).

### Controlling for Light Intensity Gradient

**Figure 3:**
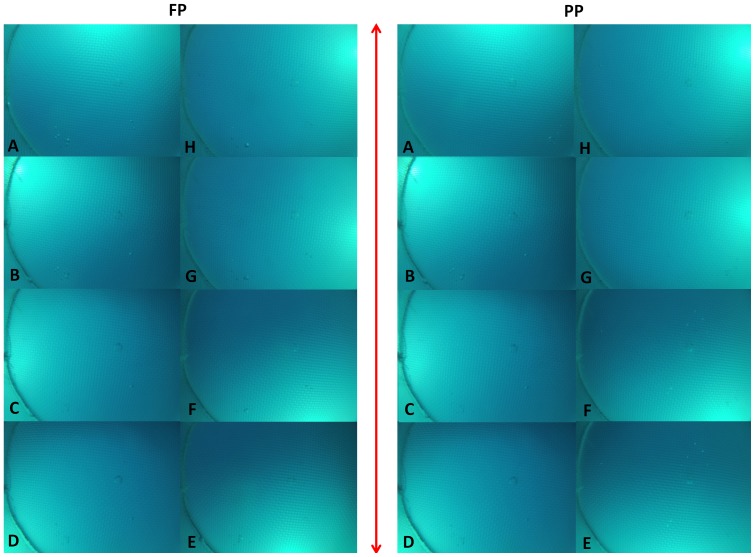
View from the behavioral chamber looking upwards, under full and partial polarization (FP and PP, respectively). The DISC was manually rotated 360° counterclockwise, at increments of ∼45° (A–H), while scuba-diving at a depth of 9 m. Red arrow indicate the filter’s e-vector orientation. Photos were taken on the 18^th^ of November 2013 at 14∶35–14∶50. Solar elevation at the time was approximately 21° from the horizon, and the sky was clear.

When the filter’s Polaroid interacts with the polarization pattern of the sky, a light intensity gradient occurs [42]. This intensity gradient was assessed using GOPRO images taken from within the behavioral chamber looking upwards, under partial and full polarization conditions (Fig. 3). The DISC was placed at a depth of 9 m and was rotated manually 360° at increments of ∼45°. Visual inspection of the photos shows that the light-intensity gradient was dominated by the direct image of the sun, with little difference in brightness or color between the two polarization conditions. A comparison of the GOPRO exposure times found no significant difference between the two conditions (2 sample t-test, t = 1.3536, p = 0.2015); indicating no difference in overall light intensity as all other camera parameters (aperture, ISO, etc.) were fixed.

### Image Analysis

Digital photographs were processed and analyzed using specialized software (DISCUS, [17]) which incorporates, among others, R [45], ImageJ (http://rsb.info.nih.gov/ij/) and EXIF Reader. Within the DISCUS framework, larval and analog compass-needle position within the picture frame are digitized and recorded as polar coordinates.

The coordinates are then used to calculate larval mean bearing and swimming directionality (R; [17], [38]). The degree of oriented swimming, or “directionality”, is quantified as the mean resultant vector of the compass-corrected cardinal coordinates (R_c_), computed from the original polar coordinates (R_o_) and the analog compass rotation. Note that R_o_ represents directionality relative to the DISC/camera-frame while R_c_ represents directionality relative to the magnetic North.

### Statistical Analysis

Larval response to polarized light in the optomotor trials was assessed using Chi-squared tests for the equality of proportions. Swimming directionality in the DISC trials was examined using the Rayleigh’s test- a goodness-of-fit test which compares the likelihood that the observed distribution of larval positions within the DISC follows a uniform rather than a von-Mises distribution [46]. Subsequent analysis of the NC experiments was limited to larvae with significant directionality (Rayleigh’s test, p<0.05) and R_c_ larger than R_o_. This criterion removed larvae with little mobility which position was not influenced by external cues [38]. The distribution of mean bearings (“among larvae”) was tested for uniformity using the Rao spacing test [46]. Both tests used the R package ‘Circular’ [47].

Regression-tree analysis (‘rpart package’ [48]) was used to examine the effect of putative predictors on the swimming directionality (R_c_) observed under naturally varying conditions. The analysis is relatively assumption free and handles complex non-linearities and interactions [49]. Screening for the optimal tree size was based on the 1SE rule, with 10-fold cross-validation and the complexity-parameter threshold set at 0.01. Ultimately, however, tree growth was limited by the ‘minimum node size’ which, given the relatively small sample size, was set to 15. The analysis excluded a single outlier (R_c_ = 0.44 with NSR = 1.00) that showed highly directional swimming, but in two opposing directions (i.e. effectively ‘canceling-out’ the high directionality in any one direction).

Rotation of the DISC while adrift resulted in the rotation of the polarization axis, imposed by the filter, relative to the sun’s position in the sky. The extent of rotation and the position of the polarization axis at each deployment differed across deployments. Assuming that larvae may respond to both polarized and non-polarization related cues (i.e. e-vector orientation and percent polarization of the filter vs. the direct image of the sun, and the brightness and spectral gradients), the discrepancy between these signals had the potential to confound the effect of percent polarization. Hence instead of analyzing swimming directionality per-se, we searched for qualitative indication that the larvae were following the rotation of the polarization axis.

## Results

### Optomotor

A total of 59 individuals were tested for their response to each of the three treatments. Of these, 57 responded to the positive BW control, 6 responded to the negative control and 39 responded to the polarization pattern (See Table S1). The proportion of positive responses elicited by the polarized cue (POL) was somewhat lower than that elicited by the BW cue, but significantly higher (proportion test, p<0.01) than the proportion expected ‘by chance’ (W). The effect of POL was significant whether borderline responses (Table S1- “B”) were ignored, counted as negative responses, or counted as positive responses (Table 1).

**Table 1:** Optomotor trials examining sensitivity of *Premnas biaculeatus* to linearly polarized light.

Chi squared	Positive to W (n = 10 borderline cases )	Positive to POL (n = 11 borderline cases)	Positive to BW (n = 2 borderline cases)	Borderline
43.9 (p<0.001)	0.12 (6/49)	0.82 (39/48)	1.00 (57/57)	Ignored
37.4 (p<0.001)	0.27 (16/59)	0.85 (50/59)	1.00 (59/59)	As positive
36.8 (p<0.001)	0.10 (6/59)	0.66 (39/59)	0.97 (57/59)	As negative

The proportion of positive optomotor responses (N = 59) to the control, polarized stripes and black-and-white stripes patterns (W, POL and BW; respectively), with different options of considering borderline cases (in which it was hard to decide whether the response was positive or negative). Ratios of fish responding with actual numbers are given in parenthesis. Chi-squared and p-values pertain to a test for the equality of proportions (proportions test); testing the null hypothesis that the probability of responding positively to POL is equal to the expected by chance (W).

### DISC – Natural Light Conditions (NC)

**Figure 4:**
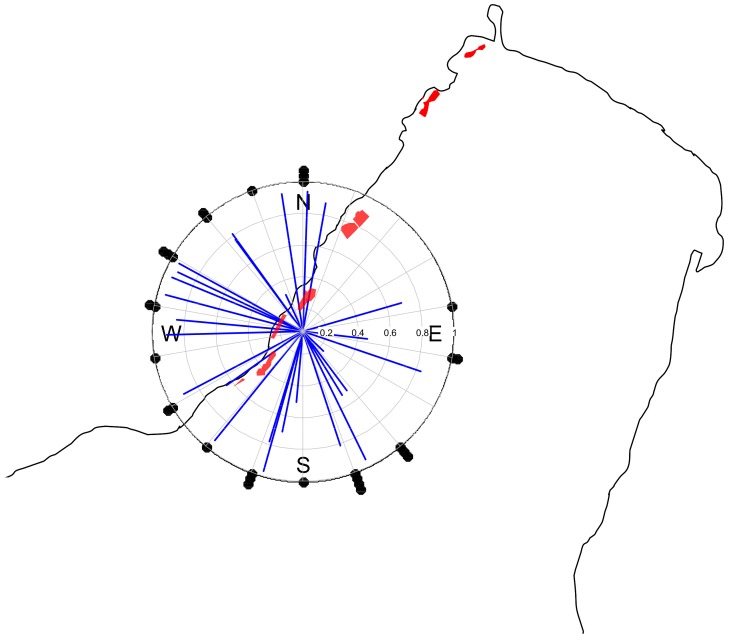
Mean bearings and directionality of *Premnas biaculeatus*. Mean bearings and directionality (R_c_) of 28 *P. biaculeatus* post-larvae, depicted by the direction and the length of the blue lines respectively. Red polygons represent nearby coral-reef patches.

R_c_ was larger than R_o_ for 67% of the post-larvae (29 of 43 post-larvae). Of these, 28 showed significant swimming directionality (Rayleigh’s test, p<0.05. Mean R_c_: 0.74±0.23), but to no particular direction with respect to the north (Fig. 4), the sun’s position, wind direction or current direction (Rao’s spacing test, p>0.1).

**Figure 5:**
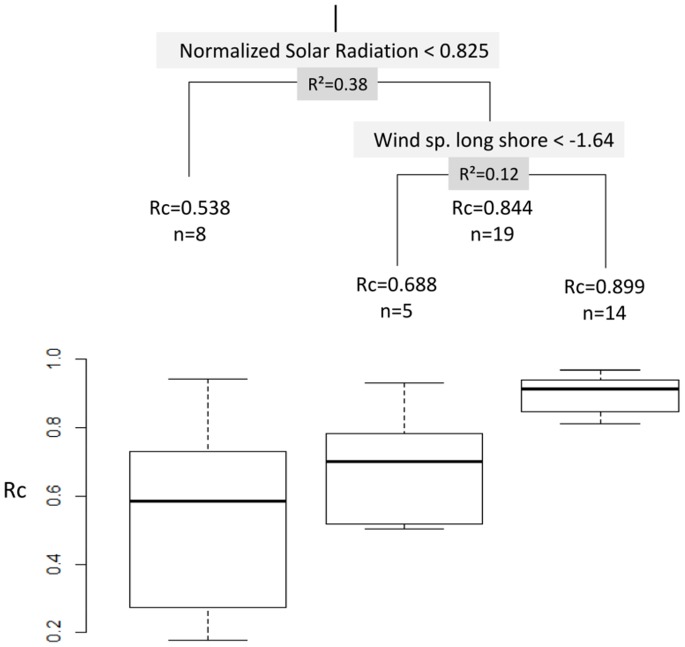
Environmental factors explaining the variation in the directionality (R_c_) of *Premnas biaculeatus*. Regression tree of swimming directionality of 28 *P. biaculeatus* post-larvae, with box plots showing the distribution of R_c_ in each terminal node.

Regression-tree analysis resulted in two bifurcations (Fig. 5). The first bifurcation indicated higher swimming directionally under clear sky (NSR>0.825), and explained 38% of the variation in R_c_. The second bifurcation split observations with NSR>0.825, associating higher R_c_ values with winds having a long-shore component greater than −1.64 m s^−1^ (negative values indicate southerly wind); or, equally, with currents having a long-shore component smaller than 4.46 cm s^−1^ (positive values indicate northbound currents). Inclusion of either variable explained a further 12% of the variance and resulted in a similar partitioning (differing on only 3 of 19 observations); which reflects an association between southerly winds with northbound water currents.

### DISC – polarized-light Manipulation

The results presented below pertain to those deployments for which either R_c_ and/or R_o_ exceeded 0.3, and |R_c_ -R_o_| was greater than 0.1. These deployments coincide with a DISC rotation greater than 80 degrees, which strengthens the signal of orientation towards either the sun or the polarization axis. Deployments with DISC rotation <80 degrees produced a far noisier response which masked the patterns of interest, and were therefore left out. The DISC rotation is indeed necessary in this case to differentiate between 2 conflicting types of cues, the rotating-polarized-cue (the filter’s e-vector orientation) and stationary, non-polarization related cues (The direct image of the sun, and the brightness and spectral gradients).

**Figure 6:**
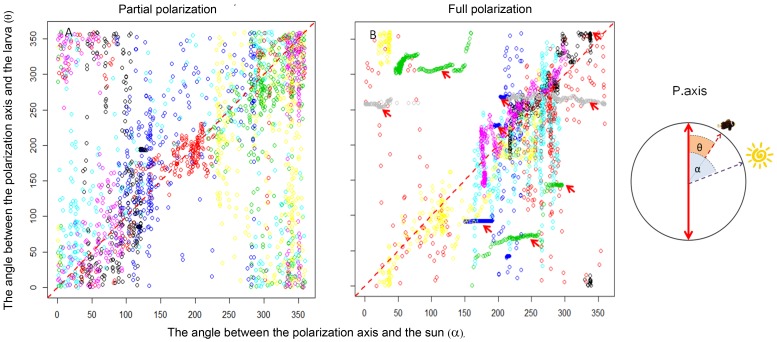
*Premnas biaculeatus* follow the rotating filter in the full polarization treatment. Larval position, measured at 2(θ; Y-axis); as the axis changes its direction relative to the sun (α; X-axis). Different colors represent different larvae, under partial and full polarization (panels A & B). Points along the equality line (dashed line) depict larvae swimming towards the sun. Horizontal sequences of points (marked by red arrows) depict larvae that track the polarization axis, as it rotates along with the DISC.

Three of eight post-larvae swimming under fully-polarized light exhibited high directionality with respect to the axis of polarization (“polarization axis tracking”; red arrows in Fig. 6). This behavior was not observed under partially-polarized light (n = 7). Moreover, the remaining four post-larvae showed higher directionality relative to the sun’s position than under partially polarized light (Points lined along diagonal in Fig. 6; Homogeneity of concentration parameters test; equal kappa test [47], p<0.001). NSR values during polarization-axis-tracking were not exceptionally different from those of the other trials (NSR values: 0.86±0.021 vs. 0.86±0.2). The experimental raw data of the DISC can be found in Table S2.

## Discussion

This study demonstrates a significant effect of sky clarity (NSR; Fig. 5) on the swimming directionality (R_c_) of post-larval *P. biaculeatus*; which we interpret as indicative of sun-compass orientation. We also show that these post-larvae are sensitive to linearly polarized-light (Table 1) and that they can orient with reference to either the sun’s position and/or to the direction of the polarization axis (Fig. 6). Yet, even the most directional post-larvae (high R_c_) varied in their mean bearing (Fig. 4). The latter is unsurprising, for two reasons. First, the need for orientation is not limited to settlement [1], [2]. Second, *P. biaculeatus* is non-native to the Red Sea and the experimental larvae were reared in captivity – i.e. they were ‘free’ of imprinting on local smells and sounds, and/or of adaptation to persistent local hydrological features; both of which have been invoked to explain orientation with respect to natal reefs [18]. Use of such ‘naïve’ larvae offers an unconfounded perspective on the response to sun-derived cues.

Our polarization-manipulation experiment presented polarized-light sensitive larvae with a rotating frame of reference (i.e. the axis of polarization, which rotates along with the DISC); resembling, in concept, an optomoter experiment. Accordingly, larvae that sought a fixed point of reference could either focus their orientation on the sun, or track the motion of the rotating polarization axis; as seen under ‘full-polarization’ (Fig. 6). A weakened rotational signal can explain the weaker ‘axis tracking ‘ behavior observed under partial-polarization, and underscores our inference that (some) coral-reef fish larvae have the potential to utilized polarized light for orientation. The difference between the two treatments (95% vs. 48%, respectively) may be indicative of the sensitivity threshold for polarized-light vision in this species. Hence, as percent polarization in natural sea-water rarely exceeds 60% [26], [32], the extent to which this cue may be of relevance remains questionable. On the other hand, it may be that given the artificial uncoupling of the sun’s position and that of the polarization axis, the larvae ‘chose’ to ignore the weaker polarization axis. Either way, the unequivocal axis-tracking under full polarization remains highly suggestive.

The polarization pattern of the sky, which is refracted through Snell’s window, is gradually lost with depth [26]. At depths greater than ∼20 m the available polarization signal is mostly due to the scattering of the downwelling light. This signal can be detected down to depths of 200 m [29] and may serve as a fixed point of reference; even if its directional information (azimuth) may be non-trivial (i.e. affected by different variables such as water turbidity, surface waves, overcast etc. [24], [25], [26]). Importantly, overcast conditions lower the percent polarization, relative to sunny conditions [50], and may thus affect not only the sun’s image and the brightness and spectral gradients, but also the polarization signal.

It is instructive to look also at the variables that contribute little, or not at all, to explaining the observed variation in R_c_ (Fig. 5). Most notable is the lack of an effect due to sun elevation; or, equivalently, time of day. Elsewhere, orientation directionality was found to follow a concave down relationship, with a minimum around mid-day ([18], Paris CB, Irisson JO, Leis JM, Boguki D, Piskozub D, et al., unpublished data). Both studies were carried at a combination of latitude and time-of-year at which the midday sun was almost directly overhead (∼90°), which provides little directional information. At the northern Gulf of Aqaba, during February- March, the midday sun is found at ∼60° above the horizon. Hence, while the sun’s elevation varies through the day, the directional information conveyed by its position remains largely unaltered; providing the sun is visible.

Larval-age and bottom-depth were also excluded from the final regression-tree model; despite a potential effect of the ontogeny on the visual system post-settlement [51], and of the bottom depth on the polarization signal [25], [28]. Further work with early pelagic larvae, from hatching to late larvae before the settlement stage is necessary. However, one possibility is that the range of values of either variable was too narrow and did not cover the critical values necessary to illicit a behavioral response. Finally, the effect, albeit small, of the longshore components of either the wind and/or the water-current may be the result of increased turbidity due to coastal upwelling (southerly wind along a western coastline). However, this explanation requires validation.

The idea of sun-compass orientation in larval fish is not new [52], [53], [54], [18] and has been demonstrated in-situ through cue manipulation (Paris CB, Irisson JO, Leis JM, Boguki D, Piskozub D, et al., unpublished data). For example, [54] reported that *Chromis atripectoralis* had significantly higher swimming directionality (R) on sunny afternoons compared with those of cloudy afternoons. They also reported that swimming directionality of *Pomacentrus lepidogenys* was higher in the morning than in the afternoon. Similarly, the idea that polarized light may aid orientation in fish has also been around for some time. For example, [55] reported that the garfish *Zenarchopterus dispar* responded to artificially induced polarization by orienting their body either perpendicular or, to a lesser extent, parallel to the axis of polarization. They also reported that the effect was significantly weaker under cloudy conditions; note however that the light-intensity gradient in that work was not controlled [42]. Our work extends these findings primarily by showing that at least some coral reef fish post-larvae can use patterns of light polarization in order to enhance their swimming directionality.

## Supporting Information

Figure S1 **The relationship between the sun’s visibility and the normalized solar radiation (NSR) index (A).** Visibility was assessed from the first photo of the first 17 deployments of the Natural Conditions (NC) experiment, taken by the DISC’s camera (B).
(TIF)

Table S1 **Experimental data of the optomotor.** Responses of *Premnas biaculeatus* post-larvae to the white, black-and-white stripes, and polarized stripes (W, BW, and POL; respectively). 0, 1 and B indicate negative, positive and borderline responses respectively.
(DOCX)

Table S2 **The experimental data of the DISC. Ro**-directionality relatively to the DISC; **Rc**- directionality relatively to the north; **NSR**-normalized solar radiation; **Mean azimuth**- larva’s mean swimming azimuth relatively to the north; **Sun’s elevation** relatively to the horizon; **Treatment: NC**-natural conditions, **PP**-partial polarization, **FP-**full polarization. For more details see methods section in the main text.
(DOCX)

Text S1 **Supporting information**
(DOCX)
