# Supplementary figures and images for: Polarized Light Sensitivity and Orientation in Coral Reef Fish Post-Larvae
Source: PLoS One. 2014 Feb 7;9(2):e88468. doi: 10.1371/journal.pone.0088468 (PMC3917914; doi:10.1371/journal.pone.0088468)

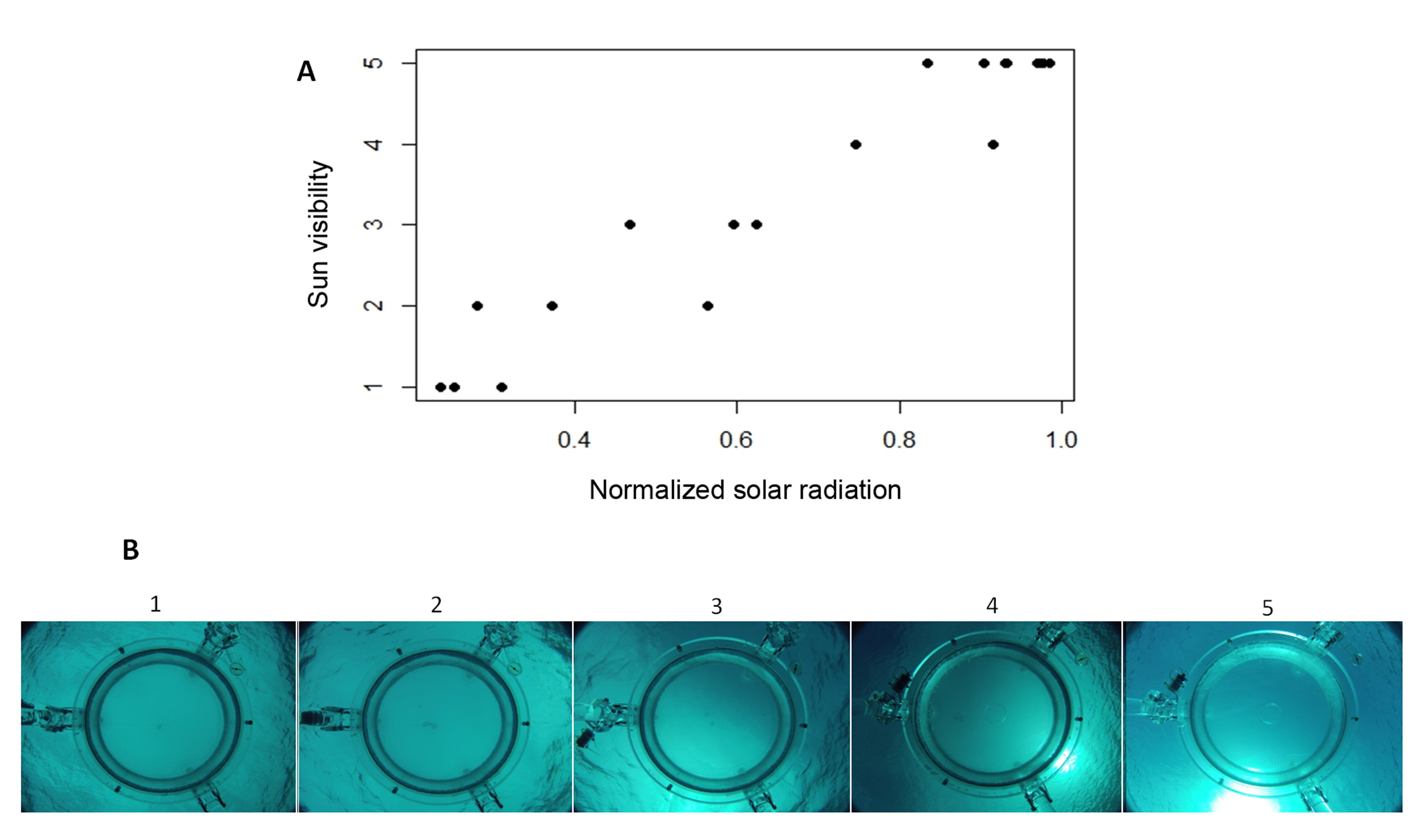

Supplement: Figure S1 — The relationship between the sun’s visibility and the normalized solar radiation (NSR) index (A). Visibility was assessed from the first photo of the first 17 deployments of the Natural Conditions (NC) experiment, taken by the DISC’s camera (B). (TIF) [file pone.0088468.s001.tif]
