# Supplementary material for: Polarized Light Sensitivity and Orientation in Coral Reef Fish Post-Larvae
Source: PLoS One. 2014 Feb 7;9(2):e88468. doi: 10.1371/journal.pone.0088468 (PMC3917914; doi:10.1371/journal.pone.0088468)
Supplement: Table S1 — Experimental data of the optomotor. Responses of Premnas biaculeatus post-larvae to the white, black-and-white stripes, and polarized stripes (W, BW, and POL; respectively). 0, 1 and B indicate negative, positive and borderline responses respectively. (DOCX) [file pone.0088468.s002.docx]

| Index # | Age | W | BW | POL |
| --- | --- | --- | --- | --- |
| 1 | 16 | 0 | 1 | 0 |
| 2 | 16 | 0 | 1 | 0 |
| 3 | 16 | 0 | 1 | 1 |
| 4 | 16 | 0 | 1 | 0 |
| 5 | 16 | 0 | 1 | 0 |
| 6 | 23 | 0 | 1 | 1 |
| 7 | 23 | 0 | 1 | 1 |
| 8 | 23 | 0 | 1 | 1 |
| 9 | 23 | 0 | 1 | 1 |
| 10 | 23 | 0 | 1 | 1 |
| 11 | 21 | 0 | 1 | 0 |
| 12 | 21 | 0 | 1 | 1 |
| 13 | 21 | 0 | 1 | 1 |
| 14 | 21 | 0 | 1 | 0 |
| 15 | 21 | 0 | 1 | B |
| 16 | 16 | 1 | 1 | 1 |
| 17 | 13 | 0 | 1 | 1 |
| 18 | 16 | 0 | 1 | 1 |
| 19 | 16 | 1 | 1 | 1 |
| 20 | 16 | 0 | 1 | 1 |
| 21 | 16 | 0 | 1 | 1 |
| 22 | 17 | 0 | 1 | 1 |
| 23 | 17 | 0 | 1 | B |
| 24 | 17 | 0 | 1 | 1 |
| 25 | 18 | B | 1 | 1 |
| 26 | 18 | 0 | 1 | 1 |
| 27 | 18 | 0 | 1 | 0 |
| 28 | 18 | 0 | 1 | 1 |
| 29 | 18 | 0 | 1 | 1 |
| 30 | 19 | B | 1 | 1 |
| 31 | 19 | B | B | 1 |
| 32 | 19 | 1 | 1 | 1 |
| 33 | 19 | 0 | 1 | 1 |
| 34 | 19 | 0 | 1 | 1 |
| 35 | 19 | 1 | 1 | 1 |
| 36 | 19 | B | 1 | 1 |
| 37 | 20 | 0 | 1 | 0 |
| 38 | 20 | 0 | 1 | 1 |
| 39 | 20 | 0 | 1 | 0 |
| 40 | 20 | 0 | 1 | 1 |
| 41 | 20 | 0 | 1 | 1 |
| 42 | 20 | 0 | 1 | B |
| 43 | 20 | B | 1 | B |
| 44 | 21 | 1 | 1 | 1 |
| 45 | 21 | 0 | 1 | 1 |
| 46 | 21 | 0 | 1 | 1 |
| 47 | 21 | 0 | 1 | 1 |
| 48 | 21 | 0 | 1 | 1 |
| 49 | 21 | 0 | 1 | 1 |
| 50 | 21 | 0 | 1 | B |
| 51 | 21 | 1 | 1 | B |
| 52 | 21 | 0 | 1 | B |
| 53 | 21 | 0 | 1 | B |
| 54 | 21 | B | 1 | B |
| 55 | 21 | B | 1 | 1 |
| 56 | 21 | 0 | 1 | 1 |
| 57 | 21 | B | 1 | B |
| 58 | 21 | B | B | B |
| 59 | 21 | B | 1 | 1 |
| Total positive |  | 6 | 57 | 39 |
