# Supplementary material for: Polarized Light Sensitivity and Orientation in Coral Reef Fish Post-Larvae
Source: PLoS One. 2014 Feb 7;9(2):e88468. doi: 10.1371/journal.pone.0088468 (PMC3917914; doi:10.1371/journal.pone.0088468)
Supplement: Table S2 — The experimental data of the DISC. Ro-directionality relatively to the DISC; Rc- directionality relatively to the north; NSR-normalized solar radiation; Mean azimuth- larva’s mean swimming azimuth relatively to the north; Sun’s elevation relatively to the horizon; Treatment: NC-natural conditions, PP-partial polarization, FP-full polarization. For more details see methods section in the main text. (DOCX) [file pone.0088468.s003.docx]

| Larva's ID | Date | Time | Ro | Rc | Mean azimuth  [deg] | Sun's azimuth  [deg] | Sun's elevation  [deg] | Larva's  Age  [days] | Solar radiation  [W/m^2^] | NSR | Wind  dir.  [deg] | Wind  Speed  [m s^-1^] | Current  dir.  [deg] | Current  speed [cm s^-1^] | Bottom depth [m] | Treatment |
| --- | --- | --- | --- | --- | --- | --- | --- | --- | --- | --- | --- | --- | --- | --- | --- | --- |
| 1 | 08/02/2012 | 12:57:36 | 0.94 | 0.89 | 229.9 | 198.6 | 43.5 | 21 | 449.5 | 0.60 | 38.5 | 8.45 | 337 | 11.68 | 125 | NC |
| 2 | 09/02/2012 | 16:19:12 | 0.26 | 0.25 | 176.0 | 243.6 | 14.3 | 22 | 70.5 | 0.37 | 24.5 | 5.8 | 37 | 20.38 | 136.5 | NC |
| 4 | 12/02/2012 | 16:04:48 | 0.67 | 0.77 | 323.9 | 242.1 | 17.8 | 20 | 96 | 0.31 | 203.5 | 3.75 | 32 | 20.04 | 77 | NC |
| 5 | 12/02/2012 | 16:19:12 | 0.22 | 0.23 | 314.0 | 245.9 | 12.9 | 20 | 44.5 | 0.24 | 208.5 | 3.6 | 131 | 16.65 | 74 | NC |
| 6 | 12/02/2012 | 16:48:00 | 0.05 | 0.06 | 166.7 | 248.9 | 8.5 | 20 | 16 | 0.25 | 210 | 2.95 | 38 | 19.86 | 72.5 | NC |
| 7 | 13/02/2012 | 10:19:12 | 0.71 | 0.83 | 108.9 | 143.2 | 38.9 | 21 | 635 | 0.92 | 34.5 | 4.45 | 32 | 9.06 | 81.5 | NC |
| 8 | 13/02/2012 | 10:48:00 | 0.82 | 0.79 | 19.5 | 155.9 | 43.8 | 21 | 706.5 | 0.93 | 58 | 4.05 | 35 | 11.03 | 78.5 | NC |
| 10 | 13/02/2012 | 12:00:00 | 0.82 | 0.82 | 179.5 | 177.3 | 47.0 | 21 | 739 | 0.93 | 81.5 | 0.9 | 44 | 12.39 | 80.3 | NC |
| 11 | 13/02/2012 | 12:14:24 | 0.72 | 0.81 | 162.1 | 186.6 | 46.8 | 21 | 705.5 | 0.90 | 145 | 0.8 | 46 | 11.83 | 83 | NC |
| 13 | 13/02/2012 | 15:50:24 | 0.62 | 0.58 | 287.2 | 240.5 | 20.2 | 21 | 145.5 | 0.47 | 207.5 | 2.1 | 39 | 19.12 | 91 | NC |
| 15 | 13/02/2012 | 16:48:00 | 0.36 | 0.53 | 149.0 | 249.2 | 8.7 | 21 | 37.5 | 0.59 | 200 | 2.75 | 52 | 21.42 | 142.5 | NC |
| 16 | 13/02/2012 | 17:16:48 | 0.15 | 0.18 | 153.7 | 252.7 | 3.3 | 21 | 3.5 | 0.28 | 222.5 | 1.15 | 56 | 19.11 | 191.5 | NC |
| 17 | 16/02/2012 | 09:36:00 | 0.89 | 0.80 | 255.3 | 137.5 | 37.0 | 20 | 368 | 0.56 | 164.5 | 1.25 | 244 | 18.91 | 95 | NC |
| 18 | 16/02/2012 | 10:04:48 | 0.79 | 0.54 | 340.4 | 144.5 | 40.7 | 20 | 432.5 | 0.62 | 331.5 | 1.45 | 252 | 14.29 | 85 | NC |
| 19 | 16/02/2012 | 10:33:36 | 0.44 | 0.64 | 234.7 | 151.1 | 43.4 | 20 | 545 | 0.75 | 297.5 | 1.65 | 256 | 9.23 | 78 | NC |
| 20 | 16/02/2012 | 10:48:00 | 0.94 | 0.94 | 355.2 | 158.0 | 45.4 | 20 | 634.5 | 0.84 | 284.5 | 1.7 | 255 | 11.31 | 73 | NC |
| 21 | 16/02/2012 | 11:16:48 | 0.87 | 0.88 | 9.8 | 165.5 | 46.9 | 20 | 769 | 0.99 | 235 | 1.35 | 250 | 9.14 | 68 | NC |
| 22 | 16/02/2012 | 11:31:12 | 0.87 | 0.77 | 313.8 | 173.7 | 47.8 | 20 | 776.5 | 0.98 | 250 | 2.05 | 248 | 8.88 | 61 | NC |
| 23 | 16/02/2012 | 14:52:48 | 0.74 | 0.78 | 196.9 | 231.8 | 30.5 | 20 | 497 | 0.97 | 256.5 | 2.7 | 118 | 2.64 | 128.5 | NC |
| 24 | 16/02/2012 | 15:21:36 | 0.64 | 0.85 | 275.4 | 237.1 | 25.5 | 20 | 403 | 0.97 | 235.5 | 1.45 | 136 | 2.58 | 157 | NC |
| 25 | 16/02/2012 | 15:36:00 | 0.66 | 0.91 | 268.7 | 241.2 | 21.0 | 20 | 334.5 | 0.96 | 311 | 2.1 | 118 | 3.46 | 167.5 | NC |
| 26 | 16/02/2012 | 16:04:48 | 0.89 | 0.94 | 292.6 | 244.7 | 16.8 | 20 | 245 | 0.90 | 341 | 1.8 | 124 | 3.24 | 182.5 | NC |
| 27 | 16/02/2012 | 16:33:36 | 0.70 | 0.94 | 299.0 | 248.0 | 12.2 | 20 | 139 | 0.91 | 332 | 2.3 | 145 | 6.00 | 222 | NC |
| 28 | 16/02/2012 | 16:48:00 | 0.55 | 0.93 | 351.3 | 251.2 | 7.6 | 20 | 62.5 | 0.98 | 177.5 | 2.7 | 119 | 7.60 | 258.5 | NC |
| 30 | 19/02/2012 | 15:21:36 | 0.94 | 0.94 | 285.1 | 238.7 | 25.4 | 22 | 259 | 0.63 | 178 | 3.85 | 334 | 2.59 | 138 | NC |
| 35 | 26/02/2012 | 12:14:24 | 0.80 | 0.81 | 324.4 | 188.6 | 51.3 | 19 | 805.5 | 1.00 | 54.5 | 3.65 | 225 | 18.73 | 82.5 | NC |
| 36 | 26/02/2012 | 12:43:12 | 0.97 | 0.97 | 195.6 | 197.4 | 50.2 | 19 | 793 | 1.00 | 59.5 | 3.65 | 229 | 30.00 | 71.75 | NC |
| 38 | 26/02/2012 | 15:36:00 | 0.94 | 0.82 | 185.7 | 243.3 | 24.4 | 19 | 405.5 | 1.00 | 53 | 3.1 | 279 | 7.32 | 82.85 | NC |
| 39 | 26/02/2012 | 16:04:48 | 0.91 | 0.94 | 1.8 | 247.0 | 19.9 | 19 | 321 | 1.00 | 51.5 | 3.6 | 226 | 8.00 | 79.5 | NC |
| 40 | 26/02/2012 | 16:19:12 | 0.27 | 0.46 | 96.5 | 250.3 | 15.4 | 19 | 205.5 | 1.00 | 46 | 3.95 | 235 | 15.00 | 76.95 | NC |
| 41 | 26/02/2012 | 16:48:00 | 0.84 | 0.92 | 295.4 | 253.5 | 10.7 | 19 | 137.5 | 1.00 | 41 | 3.95 | 230 | 20.64 | 70 | NC |
| 43 | 27/02/2012 | 11:16:48 | 0.90 | 0.95 | 154.2 | 165.7 | 51.0 | 20 | 788 | 1.00 | 36 | 2.15 | 30 | 2.82 | 86.5 | NC |
| 44 | 27/02/2012 | 11:45:36 | 0.88 | 0.90 | 242.0 | 175.1 | 51.9 | 20 | 801.5 | 0.99 | 99 | 0.6 | 15 | 3.19 | 85.5 | NC |
| 45 | 27/02/2012 | 12:43:12 | 0.71 | 0.69 | 266.8 | 201.7 | 49.7 | 20 | 771 | 0.98 | 160 | 2.95 | 22 | 6.45 | 83.5 | NC |
| 46 | 27/02/2012 | 13:12:00 | 0.83 | 0.94 | 218.6 | 208.6 | 47.9 | 20 | 738.5 | 0.97 | 145.5 | 2.95 | 22 | 7.50 | 85 | NC |
| 47 | 27/02/2012 | 13:26:24 | 0.49 | 0.52 | 143.8 | 216.2 | 45.1 | 20 | 713.5 | 0.97 | 160 | 3.35 | 31 | 7.88 | 81 | NC |
| 48 | 27/02/2012 | 13:55:12 | 0.58 | 0.70 | 191.8 | 223.0 | 41.8 | 20 | 684.5 | 0.97 | 173.5 | 3.6 | 25 | 5.96 | 76 | NC |
| 50 | 27/02/2012 | 16:48:00 | 0.46 | 0.50 | 185.3 | 255.7 | 8.0 | 20 | 75.5 | 1.02 | 183.5 | 2.65 | 44 | 11.30 | 98 | NC |
| 51 | 27/02/2012 | 17:16:48 | 0.89 | 0.88 | 255.3 | 258.5 | 3.4 | 20 | 14.5 | 1.26 | 182.5 | 2.4 | 132 | 11.23 | 108.5 | NC |
| 52 | 27/02/2012 | 17:45:36 | 0.93 | 0.93 | 285.6 | 261.2 | -1.3 | 20 | 1 | 1.00 | 209.5 | 2.45 | 45 | 9.80 | 117.5 | NC |
| 80 | 15/03/2012 | 11:16:48 | 0.99 | 0.95 | 341.1 | 163.5 | 57.5 | 23 | 807 | 1.05 | 145.5 | 0.85 | 237 | 10.25 | 68 | FP |
| 81 | 15/03/2012 | 11:45:36 | 0.26 | 0.43 | 190.4 | 179.0 | 58.6 | 23 | 831 | 1.08 | 147 | 1.3 | 233 | 8.24 | 48 | PP |
| 82 | 15/03/2012 | 12:43:12 | 0.26 | 0.80 | 219.3 | 205.9 | 55.8 | 23 | 828.5 | 1.08 | 167.5 | 1.65 | 267 | 8.53 | 81 | PP |
| 83 | 15/03/2012 | 13:12:00 | 0.92 | 0.95 | 214.2 | 215.9 | 52.9 | 23 | 812.5 | 1.12 | 169.5 | 2.4 | 259 | 7.92 | 73.5 | FP |
| 84 | 15/03/2012 | 13:40:48 | 0.14 | 0.13 | 256.8 | 227.1 | 47.9 | 23 | 765.5 | 1.13 | 133 | 2.75 | 256 | 6.84 | 80 | FP |
| 85 | 15/03/2012 | 14:24:00 | 0.63 | 0.64 | 307.1 | 233.8 | 43.7 | 23 | 692 | 1.12 | 168.5 | 2.65 | 249 | 6.58 | 93.5 | PP |
| 86 | 15/03/2012 | 16:19:12 | 0.51 | 0.66 | 269.0 | 256.0 | 19.8 | 23 | 247.5 | 1.01 | 340.5 | 3.1 | 198 | 13.37 | 147.5 | PP |
| 87 | 15/03/2012 | 16:33:36 | 0.67 | 0.95 | 257.6 | 259.3 | 14.7 | 23 | 168 | 0.95 | 335.5 | 2.55 | 166 | 10.44 | 195.5 | FP |
| 88 | 15/03/2012 | 17:16:48 | 0.12 | 0.14 | 288.5 | 263.6 | 7.7 | 23 | 30.5 | 0.60 | 340 | 2.65 | 176 | 17.57 | 130.5 | FP |
| 89 | 15/03/2012 | 17:45:36 | 0.60 | 0.54 | 168.5 | 266.7 | 2.4 | 23 | 16 | 0.76 | 352.5 | 3.6 | 194 | 12.71 | 165 | PP |
| 90 | 21/03/2012 | 09:07:12 | 0.68 | 0.68 | 0.6 | 120.3 | 42.4 | 17 | 447 | 0.62 | 29 | 8.3 | 295 | 11.47 | 91 | NC |
| 91 | 21/03/2012 | 09:36:00 | 0.59 | 0.70 | 73.7 | 128.0 | 48.0 | 17 | 486.5 | 0.63 | 33 | 9.85 | 269 | 7.17 | 76 | NC |
| 92 | 21/03/2012 | 10:04:48 | 0.30 | 0.31 | 334.8 | 135.0 | 51.9 | 17 | 545.5 | 0.65 | 34.5 | 10.05 | 286 | 3.95 | 69 | NC |
| 94 | 22/03/2012 | 12:14:24 | 0.26 | 0.35 | 180.6 | 196.9 | 60.3 | 18 | 742.5 | 0.78 | 40.5 | 8.6 | 125 | 4.08 | 49 | FP |
| 97 | 22/03/2012 | 13:40:48 | 0.32 | 0.39 | 238.0 | 226.3 | 51.8 | 18 | 685 | 0.81 | 27.5 | 8.65 | 144 | 5.32 | 85.5 | FP |
| 109 | 25/03/2012 | 10:04:48 | 0.31 | 0.58 | 170.7 | 135.1 | 54.0 | 21 | 645 | 0.75 | 39.5 | 7.05 | 0 | 9.95 | 101.5 | FP |
| 110 | 25/03/2012 | 10:33:36 | 0.74 | 0.82 | 138.9 | 144.7 | 57.6 | 21 | 671.5 | 0.74 | 41 | 6.7 | 354 | 8.97 | 86 | PP |
| 111 | 25/03/2012 | 11:02:24 | 0.06 | 0.07 | 51.5 | 155.6 | 60.4 | 21 | 698.5 | 0.75 | 40.5 | 6.25 | 344 | 8.95 | 75.5 | FP |
| 112 | 25/03/2012 | 11:31:12 | 0.10 | 0.19 | 148.3 | 168.7 | 62.1 | 21 | 711.5 | 0.75 | 42 | 5.85 | 346 | 8.56 | 62 | PP |
| 113 | 25/03/2012 | 12:00:00 | 0.73 | 0.72 | 43.3 | 187.0 | 62.4 | 21 | 711 | 0.75 | 42.5 | 5.55 | 357 | 8.51 | 82.5 | FP |
| 114 | 25/03/2012 | 12:28:48 | 0.47 | 0.48 | 243.3 | 199.9 | 61.1 | 21 | 700 | 0.74 | 40.5 | 5.1 | 358 | 8.55 | 75.5 | PP |
| 115 | 25/03/2012 | 17:16:48 | 0.30 | 0.28 | 304.0 | 267.3 | 9.2 | 21 | 54 | 0.67 | 185 | 4.8 | 26 | 5.24 | 145 | FP |
| 117 | 26/03/2012 | 13:12:00 | 0.10 | 0.10 | 242.4 | 220.8 | 56.3 | 22 | 719.5 | 0.81 | 42 | 6.3 | 358 | 5.20 | 78 | FP |
| 118 | 26/03/2012 | 13:40:48 | 0.24 | 0.51 | 201.4 | 229.5 | 52.2 | 22 | 698 | 0.82 | 42.5 | 6.2 | 312 | 5.41 | 65 | PP |
| 119 | 26/03/2012 | 13:55:12 | 0.27 | 0.29 | 284.9 | 236.0 | 48.3 | 22 | 634 | 0.81 | 38.5 | 6.1 | 288 | 4.66 | 54.5 | FP |
| 120 | 26/03/2012 | 14:38:24 | 0.25 | 0.58 | 202.7 | 243.3 | 42.4 | 22 | 562 | 0.82 | 44.5 | 6.1 | 323 | 9.14 | 82.5 | PP |
| 121 | 26/03/2012 | 14:52:48 | 0.11 | 0.19 | 10.2 | 248.2 | 37.5 | 22 | 509 | 0.81 | 44 | 5.9 | 308 | 6.60 | 74 | FP |
| 122 | 26/03/2012 | 15:36:00 | 0.55 | 0.73 | 273.3 | 255.8 | 28.2 | 22 | 376.5 | 0.82 | 40.5 | 6.3 | 335 | 5.54 | 81 | PP |
| 123 | 26/03/2012 | 16:19:12 | 0.50 | 0.84 | 24.6 | 260.4 | 21.4 | 22 | 269.5 | 0.78 | 37 | 6.7 | 359 | 6.01 | 92 | FP |
| 125 | 26/03/2012 | 17:02:24 | 0.18 | 0.17 | 2.8 | 266.8 | 10.9 | 22 | 92.5 | 0.63 | 351.5 | 3.35 | 50 | 2.78 | 87.35 | FP |
| 126 | 26/03/2012 | 17:31:12 | 0.04 | 0.06 | 214.4 | 269.8 | 5.7 | 22 | 39.5 | 1.01 | 350.5 | 3.4 | 44 | 4.07 | 88 | PP |
| 127 | 26/03/2012 | 17:45:36 | 0.05 | 0.06 | 187.0 | 272.7 | 0.8 | 22 | 4.5 | 0.47 | 344.5 | 2.3 | 59 | 3.51 | 106 | FP |
| 128 | 27/03/2012 | 10:19:12 | 0.40 | 0.33 | 192.8 | 136.9 | 55.8 | 23 | 754.5 | 0.88 | 49.5 | 4.4 | 243 | 6.76 | 96.5 | PP |
| 129 | 27/03/2012 | 10:48:00 | 0.20 | 0.35 | 144.8 | 147.6 | 59.4 | 23 | 784.5 | 0.86 | 53 | 4.95 | 241 | 8.89 | 86.5 | FP |
| 130 | 27/03/2012 | 11:02:24 | 0.79 | 0.81 | 232.2 | 158.8 | 61.7 | 23 | 785 | 0.85 | 61 | 4.6 | 242 | 10.00 | 76 | PP |
| 131 | 27/03/2012 | 11:31:12 | 0.31 | 0.32 | 191.3 | 171.4 | 63.1 | 23 | 803.5 | 0.84 | 49.5 | 4.35 | 244 | 10.24 | 66 | FP |
| 132 | 27/03/2012 | 12:14:24 | 0.53 | 0.64 | 215.5 | 191.3 | 62.9 | 23 | 838 | 0.88 | 56 | 2.5 | 242 | 6.48 | 88.5 | FP |
| 133 | 27/03/2012 | 12:28:48 | 0.50 | 0.54 | 72.4 | 203.7 | 61.3 | 23 | 742 | 0.79 | 73.5 | 2.25 | 269 | 6.96 | 80.5 | PP |
| 134 | 27/03/2012 | 12:57:36 | 0.67 | 0.92 | 197.3 | 214.2 | 58.9 | 23 | 706 | 0.77 | 86.5 | 2.1 | 211 | 13.25 | 61.8 | FP |
| 135 | 27/03/2012 | 13:26:24 | 0.11 | 0.24 | 227.6 | 226.2 | 54.4 | 23 | 786 | 0.91 | 83.5 | 3.9 | 238 | 11.63 | 47.6 | PP |
| 136 | 27/03/2012 | 13:55:12 | 0.52 | 0.55 | 200.5 | 237.0 | 48.1 | 23 | 667 | 0.85 | 118.5 | 1.75 | 257 | 4.87 | 75 | FP |
| 137 | 27/03/2012 | 14:24:00 | 0.44 | 0.44 | 234.5 | 242.7 | 43.6 | 23 | 614 | 0.86 | 180.5 | 1.7 | 216 | 6.73 | 72 | PP |
| 138 | 27/03/2012 | 14:52:48 | 0.24 | 0.89 | 266.1 | 247.4 | 39.1 | 23 | 482 | 0.77 | 210.5 | 3 | 210 | 9.24 | 70.5 | FP |
| 139 | 27/03/2012 | 15:21:36 | 0.94 | 0.95 | 227.6 | 252.2 | 33.6 | 23 | 495.5 | 0.88 | 117 | 2.8 | 214 | 8.28 | 68.25 | PP |
| 140 | 27/03/2012 | 15:36:00 | 1.00 | 0.48 | 291.4 | 256.0 | 28.6 | 23 | 384 | 0.84 | 147 | 1.35 | 215 | 8.62 | 65.5 | FP |
| 141 | 27/03/2012 | 16:19:12 | 0.12 | 0.21 | 230.8 | 260.8 | 21.5 | 23 | 287.5 | 0.83 | 311.5 | 3.05 | 152 | 4.02 | 114.25 | PP |
| 142 | 27/03/2012 | 16:48:00 | 0.31 | 0.32 | 238.8 | 265.0 | 14.6 | 23 | 182 | 0.78 | 340.5 | 2.5 | 166 | 2.48 | 142.5 | FP |
| 143 | 27/03/2012 | 17:16:48 | 0.68 | 0.67 | 93.5 | 267.4 | 10.5 | 23 | 55.5 | 0.69 | 175.5 | 2.85 | 131 | 3.41 | 152.5 | PP |
| 144 | 27/03/2012 | 17:31:12 | 0.09 | 0.04 | 224.4 | 270.9 | 4.5 | 23 | 28.5 | 0.98 | 335.5 | 2.2 | 130 | 2.41 | 171 | FP |
