# Supplementary material for: Polarized Light Sensitivity and Orientation in Coral Reef Fish Post-Larvae
Source: PLoS One. 2014 Feb 7;9(2):e88468. doi: 10.1371/journal.pone.0088468 (PMC3917914; doi:10.1371/journal.pone.0088468)
Supplement: Text S1 — Supporting information (DOCX) [file pone.0088468.s004.docx]

SUPPORTING INFORMATION

Normalized Solar Radiation (NSR):

To assess the extent by which NSR reflects the sun visibility within Snell's window, we subjectively ranked its visibility in the first photo from each of first 17 deployments; according to the scale appearing in Fig S1. Ranked visibility was significantly correlated to NSR (Spearman rank correlation: R=0.783, p<0.01). Deployments timed within 1 hour from sunset or sunrise were not considered in this analysis as the lack of sun visibility might result from the sun being too close to the horizon and outside of the camera frame.

Percent polarization:

P was calculated as:


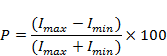


I_max_ and I_min_ are the maximal and minimal light intensities. P was calculated for wave lengths of 393-800 nm (1nm interval) of light penetrating through the three-layer filter, when a second polarized filter was placed on top of it; with e-vector orientation running parallel (I_max_) or perpendicular (I_min_) to each other. Light intensities were measured on land using a S2000, (Ocean optics) spectrophotometer.

SUPPORTING INFORMATION LEGENDS

**Fig. S1. The relationship between the sun's visibility and the normalized solar radiation (NSR) index (A).** Visibility was assessed from the first photo of the first 17 deployments of the Natural Conditions (NC) experiment, taken by the DISC's camera (B).

**Table S1: Experimental data of the optomotor.** Responses of Premnas biaculeatus post-larvae to the white, black-and-white stripes, and polarized stripes (W, BW, and POL; respectively). 0, 1 and B indicate negative, positive and borderline responses respectively.

**Table S2: The experimental data of the DISC. Ro**-directionality relatively to the DISC; **Rc**- directionality relatively to the north; **NSR**-normalized solar radiation; **Mean azimuth**- larva's mean swimming azimuth relatively to the north; **Sun's elevation** relatively to the horizon; **Treatment: NC**-natural conditions, **PP**-partial polarization, **FP-**full polarization. For more details see methods section in the main text.
